# Challenges in preventing unions and pregnancies among adolescents in rural and Indigenous communities ruled by customs and traditions in Mexico

**DOI:** 10.1590/0102-311XEN220223

**Published:** 2024-12-20

**Authors:** Lourdes Campero, Irma Romero, Fátima Estrada, Leticia Torres, Aremis Villalobos, Celia Hubert, Raffaela Schiavon

**Affiliations:** 1 Instituto Nacional de Salud Pública, Cuernavaca, México.; 2 Independent researcher, Ciudad de México, México.

**Keywords:** Indigenous Communities, Adolescent Pregnancy, Customs, Comunidades Indígenas, Embarazo en Adolescencia, Costumbres, Comunidades Indígenas, Gravidez na Adolescência, Costumes

## Abstract

Various Indigenous communities in Mexico establish their regulatory system according to their customs and traditions. In Chiapas, 27% of the population is Indigenous and has a high adolescent fertility rate. This study analyzes how customs and traditions influence unions and early pregnancies in specific contexts of rural and Indigenous communities. A descriptive qualitative research was conducted by means of semi-structured interviews with key informants who interact directly with the adolescent population. An inductive process determined analytical categories and emerging codes, forming matrices by type of informant. We interviewed 45 people (16 school staff members, 15 healthcare professionals and 14 civil society organizations leaders). The customs and traditions that characterizes these communities are linked to a traditional perception of gender relations, leading to the acceptance and promotion of unions and pregnancies at an early age. The analysis identified three situations that encourage early unions and pregnancy: clandestine dating and voluntary cohabitation without parental approval; clandestine dating and forced marital union/cohabitation agreed upon by the parents; structural and sexual violence (sale, exchange of daughters for merchandise or animals and/or rape). Adolescent pregnancy prevention strategies must address both the individual and the social and cultural context for a successful implementation.

## Resumen

Diversas comunidades indígenas en México establecen su sistema de leyes con base en sus usos y costumbres. En Chiapas, el 27% de la población es indígena y presenta un alto índice de fecundidad en la adolescencia. Este trabajo tiene como objetivo analizar cómo los usos y costumbres influyen en las uniones y el embarazo temprano en contextos específicos de comunidades rurales e indígenas. Se implementó un estudio cualitativo descriptivo mediante entrevistas semiestructuradas a informantes claves que interactúan directamente con la población adolescente. Realizamos un proceso inductivo para determinar categorías analíticas y códigos emergentes, formando matrices por tipo de informante. En total, se entrevistó a 45 personas (16 miembros del personal escolar, 15 proveedores de servicios de salud y 14 líderes de organizaciones de la sociedad civil). Las usos y costumbres que caracterizan a estas comunidades están relacionadas con una percepción tradicional de las relaciones de género, lo que conduce a la aceptación y promoción de las uniones y el embarazo a edades tempranas. Se identificaron tres situaciones que favorecen las uniones tempranas y el embarazo precoz: noviazgo escondido y convivencia voluntaria sin aprobación de los padres; noviazgo escondido y unión/cohabitación forzada por los padres; violencia estructural y sexual (venta, intercambio de hijas por mercancías o animales y violación). Para asegurar el éxito en la implementación de estrategias de prevención del embarazo en adolescentes, se debe abarcar tanto el contexto individual como el social y cultural.

## Resumo

Diversas comunidades indígenas no México estabelecem seu sistema de leis com base em seus usos e costumes. Em Chiapas, 27% da população é indígena e apresenta alta taxa de fertilidade na adolescência. Este trabalho tem como objetivo analisar como os usos e costumes influenciam as uniões e a gravidez precoce em contextos específicos de comunidades rurais e indígenas. Implementou-se um estudo qualitativo descritivo por meio de entrevistas semiestruturadas com informantes-chave que interagem diretamente com a população adolescente. Realizamos um processo indutivo para determinar categorias analíticas e códigos emergentes, formando matrizes por tipo de informante. Ao todo 45 pessoas (16 membros da equipe escolar, 15 prestadores de serviços de saúde e 14 líderes de organizações da sociedade civil) foram entrevistados. Os usos e costumes que caracterizam estas comunidades estão vinculadas a uma percepção tradicional das relações de gênero, o que leva à aceitação e promoção de uniões e gravidez em idade precoce. Foram identificadas três situações que incentivam uniões e gravidez precoces: namoro escondido e coabitação voluntária sem aprovação dos pais; namoro escondido e união/coabitação forçada pelos pais; violência estrutural e sexual (venda, troca de filhas por mercadorias ou animais e/ou estupro). Para garantir o sucesso na implementação de estratégias de prevenção da gravidez na adolescência tanto o contexto individual como o contexto social e cultural devem ser abrangidos.

## Introduction

Total fertility has decreased in Latin America and the Caribbean in the last 30 years, partly due to changes in macroeconomic factors in the region. However, adolescent fertility rates have only fallen slightly and remain the second highest globally. Studies show that illiterate adolescent girls are four times more likely to become pregnant than adolescents with secondary education. Moreover, the chances of becoming pregnant are three to four times higher among adolescents from lower socioeconomic backgrounds. Indigenous girls, particularly in rural areas, are also more likely to become pregnant at a younger age ^1^.

In Mexico, as in other Latin America and the Caribbean countries, various Indigenous communities are ruled by customs and traditions, i.e., forms of self-government or a system of collective norms, social practices, and traditions rooted in a community or social group. Practices may include common and accepted behaviors, ceremonies, rituals, social norms, and other activities. Additionally, customs and traditions are an integral part of their cultural identity that influence how they interact with each other ^2^ ^,^ ^3^ ^,^ ^4^. Community members share interests and perspectives, granting a sense of belonging and solidarity that enables their survival ^4^. The less contact communities have with the outside world, the greater their tradition is based on customs and traditions ^4^.

Each community has their own demographic differences, assimilation policies, migration movements (national and international), religious syncretism, and forms of social exclusion ^2^ ^,^ ^5^. Their problems are resolved by a council and an internal committee (health, education, and security) which influence and determine the acceptance or prohibition of any arising situation ^2^ ^,^ ^5^.

In localities governed by customs and traditions, patriarchal conceptions generate significant inequalities between men and women, an asymmetry that makes women’s development extremely fragile ^6^ ^,^ ^7^. This view is particularly concerning since it does not contemplate, and therefore undermines, the human, sexual, and reproductive rights of girls and female adolescents. Indeed, some studies highlight sexist practices, gender violence, school dropout, women buying and selling, traditional marriage patterns, forced marital unions, early motherhood, and lack of access to information and sexual and reproductive health services ^6^ ^,^ ^8^ ^,^ ^9^.

In the case of child, early, and forced marriages and unions, reports point out that they are celebrated without freedom and the full consent of at least one of the contracting parties due to coercion or social or family pressure ^10^. Even the “voluntary” marriage of minors must be interpreted with caution, as it does not always derive from a full and informed action ^4^. Additionally, child, early, and forced marriages and unions have relevant consequences in one’s integral development, including their sexual and reproductive health, since they are more likely to lead to early and unwanted pregnancies ^11^ and adverse maternal and neonatal health outcomes ^12^. In this regard, however, we must consider the youth perspective and the progressive autonomy of adolescents to make informed decisions and exercise their rights ^13^.

Hence, this work analyzes how customs and traditions influence marital unions/cohabitation and early pregnancies in rural and Indigenous communities. We specifically explore the perception and experience of key informants who work directly with adolescent population.

## Methodology

### Study site

The study was conducted in Chiapas, Mexico, where 75.5% of the population lives in poverty. Of these, 29% live in extreme poverty ^14^. More than a half of the population (51.3%) lives in rural communities, 27% are Indigenous population ^15^, 35% of married/cohabiting women are under 18 years old. Moreover, Chiapas has an adolescent fertility rate (AFR) of 84.9 live births per 1,000 women on this age group, ranking second in the country in 2020. Some municipalities in this state present an AFR higher than 300 births ^16^. The birth rate for girls aged 10-14 years is 2.75 per 1,000 girls ^17^.

We included three municipalities with a high proportion (28%) of Indigenous population from the Chiapas Highlands (San Cristobal de las Casas, Zinacantan and San Juan Chamula) ^15^. This is a unique region in Mexico, with the largest number of Indigenous Tseltsl and Tsotsil people per square kilometer, characterized by its dispersed Indigenous populations located in relatively geographically inaccessible villages. We selected the localities for convenience, considering that they had at least one lower-secondary school, a medical care unit, and civil society organizations (CSO) working with the local adolescent population. For logistical reasons, we selected sites less than two hours away from each other. To address the issues of interest for this study, we sought information variability by investigating the perspective of different actors (Box 1). This qualitative descriptive study explores the topic of interest with three social actors directly involved with youth population: school staff (SS), health personnel (HP), and CSO community leaders (CL-CSO). Using a semi-structured interview guide, we explored perceptions, opinions, and experiences around customs and traditions linked to the exercise of youth sexuality, union, and reproduction.

**Box 1:** Selection of study participants.

METHODOLOGICAL CONSIDERATIONS IN PARTICIPANT SELECTION	
School staff (SS) and Health personnel (HP)	CSO community leaders (CL-CSO)
Identification sheet collected via Google form before interviews (sociodemographic data, professional background, and training on issues regarding adolescent sexuality)	
In the selected institutions, different informant profiles were identified to achieve a plurality of experiences on the issue of interest No specific number of informants to be included per institution was previously established. The number varied according to specific institutional characteristics (infrastructure and personnel) All those related to the topics of interest were invited; informed consent was obtained, and participation was voluntary	CSO with the profile of interest were identified (who work directly in rural zones or conduct work in rural and urban zones). Identification used social networks and intentional searches on the web Leaders who conducted specific actions in study communities with adolescents were selected and invited to participate Informed consent was obtained, and participation was voluntary. Generally, one person per CSO was interviewed

CSO: civil society organizations.

Source: prepared by the authors, based on the diagnosis of sexual and reproductive health needs for preventing adolescent pregnancy in Chiapas communities, Mexico.

### Data collection and analysis

Between March 2021 and March 2022, trained personnel gathered information by means of semi-structured interviews. Given the COVID-19 restrictions, we conducted online interviews (Zoom platform; https://zoom.us).

To obtain verbal informed consent, interviewers introduce themselves as researchers from the Mexican National Institute of Public Health and explained our interest in obtaining information about their experiences and perceptions regarding adolescent pregnancy. Interviewers were trained to build rapport and understanding to encourage participants to relax and speak more honestly and openly about the discussed topics. Additionally, different topics were carefully addressed to search for patterns and possible discrepancies. All interviews were conducted in Spanish, recorded digitally and transcribed upon participant consent.

Data systematization adopted an inductive and interpretative analysis following the basic principles of Grounded Theory ^18^. We organized the information by emerging themes, based on a typology of codes and definitions created from the interview guide and adjusted throughout the coding process. For coding, we worked in triads for constant verification and discussion of the emerging preliminary findings and for consensus on code assignment ^18^. Our analysis categories included education and school permanence; sex education; dating, marital union/cohabitation, and adolescent pregnancy; risky behaviors and practices that lead to unintended or intended pregnancies; alternative life projects to early motherhood; adolescent agency, empowerment and negotiation ability. We formed arrays by type of informant to identify structural aspects and sense of the discourse. We used information triangulation between participants. We obtained data saturation on the main categories ^18^. In the end, we interviewed 45 social actors (Box 2).

**Box 2:** Participants (n = 45) by social actor.

SCHOOL STAFF (SS)			
Population profiles	Urban	Rural	Total
Teaching staff	6	4 *	10
Prefectures	2	0	2
Social workers	1	0	1
Management staff	3	0	3
Overall	12	4	16
	6 men and 10 women; average age: 42 years		
HEALTH PERSONNEL (HP)			
Hospital population profiles	Hospital	Rural	Total
Medical supervisor	1	0	1
Medical coordinator	1	0	1
Physicians	1	3	4
Nursing assistants	2 *	0	2
Medical area assistants	0	3 *	3
Community action promoter	2	0	2
Social workers	1	0	1
Psychologist	1	0	1
Overall	9	6	15
	1 man and 14 women; mostly 40-49 years old		
CSO COMMUNITY LEADERS (CL-CSO)			
Organization profile	Rural zone	Rural and urban zone	Total
Alliance with educational institutions - secondary	2 *	0	2
Alliance with health institutions	3	0	3
Broad alliance (education, health, midwifery and community)	2	7 *	9
Overall	7	7	14
4 men and 10 women; range: 19-70 years old, mean: 39 years old		

CSO: civil society organizations.

Source: prepared by the authors, based on the diagnosis of sexual and reproductive health needs for preventing adolescent pregnancy in Chiapas communities, Mexico.

* At least one of the participants was Indigenous.

Anonymity and confidentiality were assured, and verbal consent was obtained before participation. The Research Ethics Committee of the Mexican National Institute of Public Health approved the study protocol, instruments, and participant consent procedure (CI: 1,692).

## Results

### Customs and traditions internalization related to gender relations, sexuality, and reproduction

#### Gender relations

Different interviewees pointed out that patriarchal power and customs and traditions deep roots in families and communities favor gender inequalities and their normalization in daily life. According to them, men have absolute control over the body, sexuality, and reproduction: “*Women don’t even have a voice to talk about this*” (CL-CSO-2). Women cannot decide for themselves: “*...they always require the approval of their partner or even their parents...*” (HP-2). One of the teachers pointed out that women do not even leave their communities for that same reason: “*They have a more traditional thinking and are more obedient*” (SS-5). In some localities, males receive financial support from their community authorities when reaching the age of 13 years, which commits them to certain religious and community obligations and responsibilities thus favoring a self-perpetuating gender inequality from the beginning of adolescence.

#### Family-based conceptions and practices

Some communities hold the idea that “*a large family is more powerful, more respectable*” (HP-1); therefore, culturally, pregnancies are desired and promoted in general and at an early age, from 13 or 14 years. At that age, youth are considered to already have enough maturity to face pregnancy and therefore can cohabit. As soon as women menstruate, they are considered “solid” (“*maciza*”) and can marry and be mothers. Conversely, women over 20 years old are perceived as “spinsters”.

Families benefit economically when adolescents start a cohabiting relationship. Firstly, because they receive a dowry in kind or money in cash, “*it is a business to have a daughter*” (CL-CSO-9). Secondly, as the family no longer has to support their daughters, the parents are released from any commitment and “*the groom’s family takes over*” (SS-15). This implies that female youth have little chance of returning home or receiving support from their parents even when the relationship is conflictive or violent. The community too obtains an economic benefit by allowing voluntary or forced unions between adolescents since they receive a fee for granting their permission.

Once they cohabit, almost immediately pregnancy occurs. Sometimes pregnancy happens before cohabitation, but this is less common. Adolescents do not find it challenging to raise a child since “*once they grow up, they raise their little brothers*” (CL-CSO-2). In many cases, parents prohibit their young daughters from using contraceptives even after becoming mothers. However, once the first child is born, contraception is more likely to be accepted as the reproductive capacity has been fulfilled and confirmed, but it still requires the husband’s and family’s approval.

When an adolescent delivers a baby, she receives care and family support. Similarly, several communities consider people “complete” only after they have fulfilled the roles of mother-wife or father-husband, which finally gives them a voice and recognition to participate in public-community life.

Another interviewee stated that “*...in families who have enough to eat, that’s it... we think of the future, of a career... they see it as normal*” (SS-10). This type of belief and way of life is internalized from an early age, and therefore, by adolescence, people already want to form their own families. Generally, their projects materialize after finishing lower-secondary education: “*...they leave, get married or get together*” (SS-14).

#### School-based practices

The interviewees claimed that agreements are currently accepted and established in most Indigenous communities so that their population attends school at least until finishing lower-secondary education. However, some families undervalue women’s education.

“*‘There is no point for us in spending money on it’. Additionally, they see more family advantages if they drop out: …they say if you don’t want it, it’s better. Here you help me in the kitchen, with the embroidery or cutting the flowers*” (CL-CSO-15).

They commented that many parents do not see the benefit of educating their daughters “*...to seek other life opportunities*” (HP-9).

Despite this and the inadequate school infrastructure, some female adolescents do manage to study specific careers; however, most do it outside their communities. Better educated women are more empowered and have greater social participation, but on many occasions they pay the price of migrating to nearby cities at an early age and, in some instances, are unable to return to their community of origin, since they are seen as customs and traditions transgressors. Thus, it is difficult for women to have another life project beyond marital union and early motherhood.

Although customs and traditions promote union and pregnancy, the family controls and decides on dating relationships. This control becomes evident in the school environment which considered a social space that facilitates sexual approaches rejected by community norms:

“*...the* [school] *regulations are stringent when prohibiting dating relationships, and we take care that they do not happen. When we notice* [a dating relationship] *the girls tell us, ‘It’s because my boyfriend is already allowed, he already went to my house’, then we allow it because her father already agreed to it*” (SS-15).

Adolescents therefore have few spaces that favor socialization between genders, limiting their opportunities to get to know each other and establish some friendly, affective, or even sexual relationship prior to the marital union/cohabitation.

It is not uncommon for parents to ask the teaching staff to intervene and thus ensure that their daughters do not have a boyfriend, but this circumstance places teachers in an awkward position.

“*It is better that parents know their daughters have a boyfriend and thus be a little more aware... so that later they cannot complain that we did not tell them... and we avoid them saying to us ‘You knew, and you never told us’*”(SS-12).

Some families choose to stop their daughters’ formal education early and assign household chores and home care or activities with financial remuneration in favor of the family: “*...in communities with only primary school, they finish it and then girls stay at home, there is no longer contact. This distancing prevents them from marrying so young...*” (HP-9).

Conversely, in several communities governed by customs and traditions, the transmission of comprehensive sexuality education content, where rights, gender equality, and the promotion of personal projects have a primary place, is prohibited altogether. Some teachers commented that “*at school, we seek to teach something different; but at home, they learn something else. That is where we collide...*” (SS-3).

Based on these testimonies, we argue that the ideology and customs and traditions characteristic of these communities are linked to a profoundly patriarchal view of gender relations, in the family and at school, which leads to the acceptance and even the promotion of early pregnancies.

### Types of relationships and scenarios immersed in customs and traditions that make it challenging to prevent marital union/cohabitation and early pregnancy

According to the different interviewees, three situations favor early unions.

#### Clandestine dating relationship and voluntary union without parental authorization

In more traditional communities, adolescents form dating relationships clandestinely when families do not agree upon courtships. They may sometimes agree to the union disregarding their parents’ opinion either as a response to family problems or because they perceive security in what their partner promises them.

In these cases, the women escape with a man who can be much older, “*sometimes 30 or 40 years old... the families have many problems, so adolescents see an escape with a man who gives them security, even though they are much older*” (SS-11). Is unnecessary for the family to engage them in marriage; they come together by themselves and, generally, if they were studying, they drop out. Parents say, “*I’m not sending you to school to get a boyfriend*” (SS-15).

#### Clandestine dating relationship and forced union agreed by the parents

When adolescents maintain a clandestine relationship and are discovered by their relatives or someone from the community, they have to face the consequences given the community agreement that young people must complete lower-secondary education and “*when an adolescent leaves school due to pregnancy, union or work, parents have to pay a fine to the school*” (SS-1). In some instances, community authorities and the parents also impose a sanction: “*They can be fined or have the boy sent to jail*” (HP-10). Additionally, the couple’s union is made mandatory, especially if they suspect they had sexual intercourse. Importantly, a forced union can occur just because girls are seen talking with a man, even if it is just a rumor, regardless of age or consent.

“*I had the case of a first-year student with a third-year student. They got married because their parents saw them in a motorcycle taxi and sent for them from the* [municipal] *president* [local authority]*. On Monday they did not show up to school because they were already married. Two years later they separated, and the girl was left with two children and she did not finish lower-secondary*” (SS-14).

Some mention that adolescents have no right to exercise their sexuality outside marital union/cohabitation without facing social consequences. “*Unwanted pregnancies begin there. They only had* [sexual] *relations, they did not want to get married*” (HP-10). Interviewees mentioned that, in a few cases, “*if the girl becomes pregnant, parents do not force them to cohabit; but they do force them to support the baby, to pay alimony*” (HP-3).

#### Structural and sexual violence

The custom of selling or exchanging girls and adolescents for merchandise or animals still persists since “*their authorities allow making pacts between families*” (HP-1). Once they begin to menstruate, “*girls are* [marital union/cohabitation] *candidates*” (HP-3). Some parents even “*betroth their daughters from a young age, and when they reach a certain age, an agreement is made for them to leave with the boy*” (SS-5).

This is linked to the strict surveillance of women’s virginity and the “commercial” value attributed to it: parents take care that they do not associate with men until there is a betrothal or payment for the bride. Since women are not granted rights, there is no question of whether they want to get married.

“*A boy can decide for her. He goes to the authority and tells them ‘I want to get married because she was talking to me’, and the girl has to get married... that’s how they ensure women’s virginity because if she leaves the community to go study, they no longer know if she’s still a virgin*” (CL-CSO-13).

In turn, men are taught that after finishing primary or lower-secondary education they must work to buy their wives. Sometimes they migrate temporarily to raise money. “*They are not forced; they are happy because in the future, they are going to buy their wife*” (HP-11).

“*If the boy liked her, there are always women for sale. The younger, the more expensive... 13, 14-year-olds cost more money. When they get together, money is given to the girl’s parents. Sometimes the* [older] *man has another woman, but he likes the teenager... Many men have two to three wives, sometimes even sisters; in some situations, they even live in the same house*” (HP-12).

Interviewees also report that since men in the family and the community are the ones afforded rights over women, sexual violence and rape are accepted and normalized facts. Thus, rather than initiate a complaint process, reparation for the damage is solved through a marital union or economic compensation, mainly when pregnancy occurs. “*Unfortunately, adolescents are under the authority of their parents and the regional customs and traditions, so they remain silent... later, it comes out that there is a pregnancy due to rape*” (HP-4).

Less extreme cases, which also reflect a constant abuse of authority, are when adolescent couples get together and are pressured by their parents and in-laws to get pregnant soon. “*Whether or not they want to have a baby, they feel obligated* [to have children] *to maintain the marriage*” (CL-CSO-13). This is how the marital/cohabiting relation is socially consolidated.

Despite these types of relationships and scenarios, some adolescents have begun to confront behaviors and attitudes rooted in their communities’ customs and traditions due to migration processes (local, national, or international) to study or work, since they are exposed to other environments. This leads them to “*no longer accept what is indicated in their communities; they reject it*” (HP-7), to question certain practices or become open to acquiring new information that modifies their customs and traditions. “*Now that they are more informed, they make better decisions. Another fundamental piece for change is technology, the use of the Internet and cell phones*” (HP-2). Some interviewees perceive that the latter is influencing even Indigenous population; however, they note that changes are slower in these cultural contexts. Additionally, some participants mentioned the benefit of the work that some CSOs, school personnel, and health providers have conducted regarding sexual education.

## Discussion

This research analyzed the perceptions and experiences of key informants who work directly with adolescents in Indigenous communities. From their narratives, we evinced the different ways customs and traditions influence child, early, and forced marriages and unions and early pregnancy in their communities.

From the perspective of sexual and reproductive health, in particular adolescent pregnancy and its prevention, our analysis showed that given the nature and roots of customs and traditions, early reproduction is not perceived as an issue; on the contrary, it is upheld and reinforced as socially expected according to the set of beliefs and community organization. Other studies in Chiapas or with Indigenous populations show similar evidence ^16^ ^,^ ^19^ ^,^ ^20^.

Results show that the patriarchal structure and power are naturalized in customs and traditions. As mentioned in other studies, this generates significant disadvantages between men and women ^7^ ^,^ ^8^ ^,^ ^19^. We also found gender and age asymmetry between couples in different narratives, and many examples of how the youth integral development is hindered. Normalized inequality violates their human rights in their daily lives. The devaluation of women is naturalized to the point of making their individual needs invisible. Testimonies repeatedly show how the symbolic and practical construction that women exist according to the needs of men is maintained: that she is a good with “commercial” value dependent on age and virginity, and whose meaning of life depends first on their role as daughter and immediately after as wife, mother/reproducer, responsible for unpaid work essential for taking care of men, children, and the community ^4^ ^,^ ^20^ ^,^ ^21^. We found multiple family and community resistances towards a cultural change that would allow women, from childhood to adolescence, to develop critical thinking and life projects beyond early union and motherhood.

Education leads to individual improvement and social progress by enhancing physical, emotional, intellectual, and interpersonal capacities that favor the integral development of both men and women ^22^. Despite being a recognized right and constitutional obligation for all individuals ^21^, for Indigenous youth school access or permanence is not guaranteed. For the participating communities, even the school is perceived as a threatening environment since it is a source through which adolescents acquire sexual education and because it opens up the possibility of establishing friendly, affective and even dating relationships.

Other research has shown that the greater the educational gap, the greater the number of child, early, and forced marriages and unions and adolescent pregnancies. This is relevant in contexts such as Chiapas, which ranks first in Mexico in educational lag ^23^, where four out of ten women aged 15 to 19 do not attend school ^24^, where the percentage of marriages before the age of 18 is 45% ^4^, and which has one of the highest rate of adolescent pregnancies in the country ^16^.

Even when adolescents stay in school, our results show that in these communities, formal education limits the transmission of substantive topics about health in general and sexual and reproductive health in particular. Not even basic issues about the human body and its reproductive function are taught in school, and talking about pleasure as part of a healthy sexuality is completely left out. All this diminishes youth’s ability to understand the world more broadly, to acquire a minimum of financial independence, and to make decisions in different areas of their life ^25^.

In urban populations, early unions usually derive from pregnancy; however, in rural and Indigenous communities, marital union/cohabitation among adolescents precedes pregnancy, a situation that has also been documented in other studies ^9^. We found that these unions can be encouraged or allowed by customs and traditions; they can even be voluntary, but are also forced by sale or rape. These situations violate women’s rights, and they inevitably lead to pregnancy at an early age, depriving adolescent girls of having better and greater personal development and harming their health and autonomy ^19^ ^,^ ^20^ ^,^ ^25^. Notably, although adolescents in voluntary unions may choose a marital relationship, it is ultimately customs and traditions that guide and limit their decision-making. We must therefore reflect on the real possibilities that these adolescents have to progressively exercise their autonomy in vulnerable contexts where deep-rooted customs and traditions exist. Moreover, child, early, and forced marriages and unions are a clear violation of adolescents’ fundamental human rights and of their autonomy when choosing a partner and desired pregnancy. We must provide adolescents with tools that help them fully exercise their sexual and reproductive rights, including the choice of motherhood and paternity, as well as to prevent unintentional unions and pregnancies ^26^.

As our findings show, preventing child, early, and forced marriages and unions and adolescent pregnancy in rural and Indigenous communities that have entrenched customs and traditions represents a significant challenge for the state, community, family and individuals. Adolescent pregnancy prevention strategies must continue to be supported by public policy under a rights-based approach, and given its complexity, multiple sectorial and intersectorial actions will be needed. Until now, the impact that different social actors have had with parents and adolescents has been gradual and local. However, strengthening these strategies at the population level through specific programs could help modify conservative patterns that are deeply rooted in the population.

One cannot disregard the argument and weight given to family alliances and the need and role for early unions to support families’ subsistence and preservation of the ethnic group in impoverished contexts ^19^. Even when these two facts are greatly valued in Indigenous communities, they cannot justify the systematic violations of women’s and young women’s rights. Likewise, community reflection is necessary so that education and social mobility of women is recognized as a right that generates changes for the benefit of the individual, families, and society instead of being perceived as a transgression of the customs and traditions.

In this regard, the fundamental rights of individuals and of Indigenous identities are prioritized in Mexico. In its second article, the *Federal Constitution* states that customs and traditions are recognized as forms of self-government ^27^, and that the communities should enjoy reinforced protection given their vulnerability and cultural identity ^28^. However, the normative systems of Indigenous peoples cannot go against the fundamental rights of individuals, even less those of children and adolescents, leaving them subject to community agreements. In other words, in matters regarding childhood and gender, the *Convention on the Rights of Children* emphasizes that cultural practices cannot be justified if they are detrimental to their dignity, health, or development ^16^.

Recent federal efforts to advance gender equality and the women’s rights agenda included raising the legal age for marriage to 18 years ^29^ and adopting the draft decree on forced cohabitation of people under 18 to reform the *Federal Criminal Code*. This draft includes a definition of forced cohabitation of people under 18, either with or without the parties’ consent, and punishable by up to 15 years in prison and a monetary fine ^30^. Although both legal modifications are necessary, they have been insufficient and inadequate to prevent female youth’s rights violations and to promote the development of a dignified life, free of violence - a life that, among other issues, can help avoid child, early, and forced marriages and unions and early, unwanted and forced maternity. Conversely, these laws can have adverse, symbolic, and practical effects, undermining adolescents’ sexual and reproductive autonomy by not recognizing their ability to decide when to initiate sexual relationships or marital unions/cohabitation voluntarily and consensually. Additionally, there is a risk of criminalizing Indigenous communities where these practices are more frequent ^31^. It is important to consider respect for the social and cultural differences of Indigenous peoples and their community rights by restoring positive cultural practices and challenging those that hinder the comprehensive development of adolescents and their communities ^32^.

Continuing efforts are necessary so that local regulatory systems and the exercise of human rights, including the sexual and reproductive rights of adolescents, align and focus on ensuring this population’s health, education, and well-being. Community alliances between schools, health services, parents, and civil society are necessary to sensitize the population. It is also essential to involve individuals who play a key role in the communities, such as grandmothers or mothers, in designing and implementing strategies, since these have shown favorable results in reducing child, early, and forced marriages and unions and adolescent pregnancy ^33^. It is critical to explain the advantages of formal education, without gender discriminations and gaps, of providing comprehensive sexuality education, including gender perspective, and the approach to new masculinities ^17^ ^,^ ^34^. Additionally, expanding access to safe abortion services and improving contraceptive counseling is vital ^35^. Comprehensive sexuality education and access to services would allow to shift to new norms and new practices thereby improving adolescent sexual and reproductive health.

Additionally, other actions that can be taken include training teachers and health providers to safely teach comprehensive sexuality education ^17^. Promoting and disseminating the benefits of comprehensive sexuality education among adults who are directly involved in youth education would favor a better understanding of its importance. Among the key benefits are the postponement of sexual debut, a reduced number of sexual partners, and the reinforcement of preventive and responsible practices. Another strategy to be strengthened, which has been shown to be effective in this population, is the dissemination of culturally relevant information by peers through CSOs ^19^ ^,^ ^20^.

A study limitation to be acknowledge is data collection during the COVID-19 pandemic and interviewing using digital platforms. Despite implementing rigorous data collection procedures to obtain homogeneous information, we recognize potential bias as some people could feel intimidated. Currently, the use of information and communication technologies is an accepted and valid method for collecting data in research studies. An advantage posed by technology is that information is obtained quickly and in real-time. In the case of online interviews, we can maintain data integrity without losing the quality of in-person interactions between informants and researchers ^36^.

In conclusion, to ensure and increase youth well-being the cultural health approach must consider two inter-related dimensions: the individual factors and the social system in which they are embedded ^32^. The new generations must have their access to education, health, a life project and a dignified life free of violence secured. With this, the fundamental human rights of universality, interdependence, indivisibility, and progressivity would be fulfilled ^37^.
